# Keep S’Myelin: World Brain Day 2021 Editorial for Residents and Junior Doctors Page—Neurology International MDPI

**DOI:** 10.3390/neurolint13030039

**Published:** 2021-08-12

**Authors:** Lakshini Gunasekera, Tissa Wijeratne

**Affiliations:** Department of Medicine and Neurology, AIMSS, Melbourne Medical School, University of Melbourne and Western Health, Sunshine Hospital, St Albans, Melbourne, VIC 3021, Australia; lakshini_slc@hotmail.com

Every five minutes someone in the world is diagnosed with multiple sclerosis [1]. This is usually a young female in the prime of their life with a mean age of 32 years of age [2,3]. Once diagnosed, everything in their life stops for a moment. All plans for education, career, travel and relationships stop. Time stops. This year, the World Federation of Neurology (WFN) and Multiple Sclerosis International Federation (MSIF) shine the spotlight on multiple sclerosis on World Brain Day this 22 July. The message is simple—let’s stop multiple sclerosis before it has a chance to stop us.

Each year, the WFN selects an important neurological condition to focus on for the year, in collaboration with another global organisation. This year, WFN has united with MSIF to shed more light onto multiple sclerosis and advocate for better brain health globally [4,5,6]. This inflammatory disease has multiple contributory causes, ranging from one’s geographic location, low vitamin D levels to insidious viral insults. There is ultimate damage to myelin which is a protective covering wrapped around nerves to provide faster cell-to-cell communication within the central nervous system. Common presenting symptoms include double vision, loss of vision and pins and needles. MRI scans of the neuraxis can be useful in both diagnostics as well as determining the extent of radiological disease activity. Fortunately, treatments have increased significantly recently so patients are generally able to select from oral, injectable and infusional therapy. It is worth noting that 70% of countries struggle to secure an early diagnosis or quality treatment for MS as of 2021 [2]. We have come a long way but for a disease without a cure as yet, there is also still a long way to go.

World Brain Day is not just about raising awareness about multiple sclerosis existing. It is about advocating for global change to allow better education, access to early evaluation, timely investigations and equitable delivery of therapeutics. Multiple sclerosis cannot be taken at just face value. It may not be as obvious as a young woman with a wheelchair or a walking stick. Pain, fatigue, declining cognition, spasms, depression, sexual dysfunction and bladder/bowel dysfunction are some of the hidden or invisible aspects of multiple sclerosis we want to raise awareness about this year. We hope that this day is the start of a year-long campaign to shed light on this condition for education purposes, to generate more research into diagnostics and therapeutics, provide resources to help in daily clinical practice and offer better access to treatments globally. 

How can neurology residents get involved? You can help by visiting the world brain day website (https://wfneurology.org/world-brain-day-2021) (accessed on 10 August 2021) and by downloading the World Brain Day toolbox. Support the 2.8 million MS patients worldwide by joining social media campaigns to promote and endorse World Brain Day and improved brain health. You are warmly invited to download the tool box contents and share on your Facebook, twitter, LinkedIn and Instagram regularly.

World Brain Day combines the efforts of neurologists, neurological scientists, allied health staff, advocacy bodies and patient support groups to stop multiple sclerosis. However, the younger generation of doctors needs to carry this momentum forward. This is why all of us—as junior doctors and future leaders in patient advocacy—must step up to promote World Brain Day activities. It starts with sharing these key themes on social media and utilising resources available on the WFN website in day-to-day clinical practice. Let’s put an end to multiple sclerosis, starting on World Brain Day. It’s only then that we can all keep s’myelin.
